# Association between dietary total antioxidant capacity and chronic obstructive pulmonary disease: findings from the national health and nutrition examination survey

**DOI:** 10.1186/s12890-026-04595-3

**Published:** 2026-08-13

**Authors:** Huaiyong Tan, Yu Wang, Limin Wu

**Affiliations:** Department of Pulmonary and Critical Care Medicine, Suzhou Guangci Cancer Hospital, Suzhou, 215000 China

**Keywords:** Antioxidants, Chronic obstructive pulmonary disease, Cross-sectional studies, Diet, NHANES, Oxidative stress

## Abstract

**Background:**

This study aimed to investigate the association between dietary total antioxidant capacity (TAC) and chronic obstructive pulmonary disease (COPD) using data from the National Health and Nutrition Examination Survey (NHANES).

**Methods:**

This cross-sectional study included participants aged ≥ 40 years from NHANES cycles 2001–2018. COPD was defined as a self-reported physician diagnosis. TAC was calculated from 24-hour dietary recall data and categorized into quartiles. Weighted multivariable logistic regression models were used adjusting for demographic, socioeconomic, behavioral, and clinical factors to assess the association between TAC quartiles and COPD. Restricted cubic spline (RCS) analysis was performed to explore the dose-response relationship, and subgroup analyses were conducted to evaluate effect modification. Inverse probability weighting and multiple imputation were used for sensitivity analyses.

**Results:**

A total of 29,225 participants were included in the final analysis, of whom 2,829 (9.7%) had COPD. In the fully adjusted model (Model 3), higher TAC was associated with lower odds of COPD compared with the lowest quartile (Q1): Q2 (OR = 0.83, 95% CI: 0.66–1.02, *P* = 0.084), Q3 (OR = 0.71, 95% CI: 0.57–0.88, *P* = 0.003), and Q4 (OR = 0.78, 95% CI: 0.62–0.99, *P* = 0.040). RCS analysis demonstrated a significant nonlinear inverse dose–response relationship between dietary TAC and COPD risk (P for overall = 0.005; P for nonlinearity = 0.024). No significant effect modification was observed across any of the examined subgroups (all P for interaction > 0.05). Sensitivity analyses using inverse probability weighting and multiple imputation confirmed the robustness of the results.

**Conclusion:**

Higher dietary antioxidant capacity is associated with lower COPD risk in a nonlinear manner independent of smoking intensity, warranting further longitudinal research to confirm these findings.

**Clinical trial number:**

Not applicable.

**Supplementary Information:**

The online version contains supplementary material available at https://doi.org/10.1186/s12890-026-04595-3.

## Background

Chronic obstructive pulmonary disease (COPD) is a chronic respiratory condition characterized by persistent and progressive airflow limitation, representing a major global cause of morbidity, disability, and mortality [1]. According to the Global Burden of Disease Study 2021, COPD affected approximately 213.4 million people and was responsible for approximately 3.7 million deaths worldwide in 2021, with an age-standardized prevalence rate of 2512.9 per 100,000 population, imposing a substantial disease burden [2]. Pathophysiologically, COPD involves persistent inflammation of the airways and lung parenchyma. This inflammation is typically triggered by long-term exposure to harmful particles or gases, such as tobacco smoke [3]. Given its high prevalence and poor prognosis, identifying modifiable risk factors—such as smoking and environmental exposures—is crucial for the primary prevention of COPD.

Oxidative stress plays a central role in the development and progression of COPD [4]. Reactive oxygen species (ROS) from exogenous sources (e.g., cigarette smoke, air pollutants) and endogenous processes can overwhelm the lung’s antioxidant defense system, leading to oxidative damage. This damage exacerbates inflammatory responses (e.g., promoting neutrophil infiltration and pro-inflammatory cytokine release), induces protease-antiprotease imbalance, causes mucus hypersecretion, and directly injures lung parenchymal cells, which collectively drive disease progression and acute exacerbations [5, 6]. Furthermore, oxidative stress is closely linked to systemic inflammation and common comorbidities in COPD patients, such as cardiovascular disease [7]. Therefore, enhancing the body’s antioxidant defenses, particularly through dietary means, may help mitigate the risk of COPD.

Regarding dietary factors, dietary total antioxidant capacity (TAC) is a composite measure that assesses the total antioxidant potential of all antioxidant components in the diet, including vitamin C, vitamin E, carotenoids, and polyphenols [8]. Compared with studying single nutrients in isolation, TAC provides a more comprehensive reflection of the overall antioxidant effect of the diet and may capture synergistic or additive interactions among different antioxidants [9]. Previous studies have shown that higher dietary TAC is associated with reduced risks of cardiovascular disease, type 2 diabetes, and certain cancers [10–12]. Also, previous studies of individual antioxidants and antioxidant-related dietary exposures have suggested potential benefits for respiratory health. However, direct evidence regarding composite dietary TAC remains limited. An Italian population-based study found that higher dietary TAC, assessed using the ferric reducing antioxidant power method, was associated with better FEV₁ and FVC, particularly among premenopausal women who had never smoked [13]. Similarly, a UK cohort study examining processed meat, fruit and vegetable intake, and dietary TAC reported associations between dietary oxidative balance and lung function [14]. More recent studies using composite dietary antioxidant indices have also observed inverse associations with COPD prevalence, although differences in study populations, antioxidant indices, outcome definitions, and confounder adjustment have produced some variation in the findings [15, 16]. Further, it should be noted that TAC is a composite dietary metric derived from food composition databases rather than a direct biomarker; its accuracy depends on the completeness and validity of the underlying nutrient databases and the precision of dietary recall instruments.

The National Health and Nutrition Examination Survey (NHANES) is a large-scale, nationally representative survey that provides detailed dietary intake data, comprehensive health status information, and a large nationally representative sample [17]. Although NHANES includes spirometry data in select cycles, the availability of self-reported physician-diagnosed COPD across all included survey cycles (2001–2018) allowed for greater sample size and statistical power. This offers a suitable database for in-depth exploration of the potential association between dietary TAC and COPD. Based on this, the present study aimed to utilize NHANES data from 2001 to 2018 to evaluate the association between dietary TAC intake and the risk of COPD among U.S. adults, investigate its dose-response relationship, and explore effect heterogeneity across different population subgroups, thereby providing scientific evidence for dietary interventions in COPD prevention. We hypothesized that higher dietary TAC would be inversely associated with the prevalence of COPD in a nationally representative adult population, and that this association would exhibit a nonlinear dose-response pattern.

## Methods

### Study design and population

This cross-sectional analysis used data from eight consecutive cycles (2001–2002 to 2017–2018) of NHANES. NHANES is conducted by the National Center for Health Statistics (NCHS). It employs a complex, multistage, stratified probability sampling design to obtain a representative sample of the non-institutionalized civilian U.S. population. Furthermore, the NCHS Research Ethics Review Board approved all survey protocols, and all participants provided written informed consent. This study does not require additional ethical approval. No additional institutional review board approval was required for the present secondary analysis of de-identified, publicly available NHANES data.

All participants from nine consecutive NHANES cycles (2001–2002 through 2017–2018) were initially screened. The following exclusion criteria were applied: (1) age < 40 years; (2) missing information on COPD status; (3) missing data required for dietary TAC estimation.

### Assessment of COPD

COPD status was determined based on self-reported physician diagnosis. From the Medical Conditions questionnaire, participants were classified as having COPD if they answered “yes” to any of the following questions: “Has a doctor or other health professional ever told you that you had COPD?“; “Has a doctor or other health professional ever told you that you had chronic bronchitis?“; or “Has a doctor or other health professional ever told you that you had emphysema?“. This questionnaire-based definition of COPD has been widely used and validated in NHANES-related studies [18, 19].

### Measurement of dietary TAC

Dietary TAC was estimated based on data from the first 24-hour dietary recall interview. A comprehensive database containing the antioxidant capacity of various foods was used to calculate the TAC value for each reported food item [20]. The TAC was calculated using the following formula:

$$\begin{aligned}TAC=\\&\sum\:\left[Antioxidantcontent\right(\frac{100g}{d})\\&\times\:Antioxidantcapacity(\frac{mgVCE}{100g}\left)\right]\end{aligned}$$

VCE denotes vitamin C equivalents.

After calculating individual TAC values, the TAC values of all food items were summed to derive the total daily dietary TAC (mg VCE/day) for each participant. For analytical purposes, TAC was categorized into four quartile groups (quartiles Q1 through Q4) based on the distribution of TAC values in the study population, with Q1 representing the lowest intake group. Natural log-transformed TAC (lnTAC) was used for dose-response, and subgroup analyses.

### Covariates

A comprehensive set of covariates was included to account for potential confounding in the association between TAC and COPD, encompassing demographic, socioeconomic, behavioral, clinical, and nutritional factors. Demographic variables included age, sex, and race/ethnicity. Socioeconomic characteristics comprised education level, family poverty-to-income ratio (PIR; categorized as low [< 1.3], middle [1.3–3.49], or high [≥ 3.5]) [17], and marital status. Health behaviors and health status variables included smoking status (never, former, current), alcohol consumption (never, former, mild, moderate, or heavy), body mass index (BMI, kg/m²), and cumulative smoking exposure quantified as pack-years. Pack-years were calculated as the average number of cigarettes smoked per day divided by 20, multiplied by the number of years of smoking. Smoking duration was derived from the age at initiation of regular smoking to the current age for current smokers and to the age at smoking cessation for former smokers; never-smokers were assigned zero pack-years. Comorbidities were defined as self-reported physician diagnoses of hypertension, diabetes, cardiovascular disease (including coronary heart disease, heart failure, or stroke), and asthma. Total energy intake (kcal/day) and total physical activity (MET-min/week) were included as continuous covariates in the fully adjusted models.

### Statistical analysis

All analyses accounted for the complex, multistage sampling design of NHANES and incorporated appropriate sampling weights, stratification, and clustering variables. Weighted analyses were conducted using the survey package in R (version 4.5.0), with the dietary day-one sample weight adjusted for combining multiple survey cycles. Continuous variables are reported as weighted means with standard errors (SE), and categorical variables as weighted percentages with unweighted frequencies. Group comparisons were assessed using Rao–Scott chi-square tests for categorical variables and Kruskal–Wallis tests for continuous variables. Weighted multivariable logistic regression was used to evaluate the association between TAC (categorized into quartiles) and COPD. Three sequential models were constructed: Model 1 (crude); Model 2 adjusted for demographic and socioeconomic factors (age, sex, race/ethnicity, education, PIR, marital status); Model 3 (fully adjusted) additionally included smoking status, alcohol use, body mass index, comorbidities (hypertension, diabetes, cardiovascular disease, asthma), total physical activity, total energy intake, and pack-years of smoking. McFadden-like pseudo-R² and Tjur R² were reported for each model to evaluate explanatory strength. The primary analysis used a complete-case approach; sensitivity analyses using inverse probability weighting (IPW) and multiple imputation for missing covariates were conducted to assess the robustness of the results. Furthermore, a comparison of baseline characteristics between participants with and without available TAC data was performed to evaluate whether missingness was related to key covariates. A *P* for trend was derived by modeling the median TAC value of each quartile as a continuous variable. A restricted cubic spline (RCS) analysis with four knots (at the 5th, 35th, 65th, and 95th percentiles) was performed to examine the dose–response relationship between natural log-transformed TAC (lnTAC) and COPD risk. Likelihood ratio tests evaluated overall association significance and nonlinearity. Subgroup analyses were conducted by stratifying the population by age, sex, race/ethnicity, education, income, marital status, smoking, drinking, and comorbidity status. Within each stratum, the association between a 1-unit increase in lnTAC and COPD was assessed using the fully adjusted Model 3. Interaction terms between lnTAC and each stratification variable were tested to evaluate effect modification. All statistical analyses were performed using R software (version 4.5.0). All statistical tests were two-sided, and a P value < 0.05 was considered statistically significant.

## Results

### Study sample and baseline characteristics of COPD and non-COPD groups

A total of 91,351 participants from NHANES 2001–2018 were initially considered. After excluding 58,292 participants (58,284 aged < 40 years and 8 with missing COPD data) and 3,834 participants with missing dietary TAC data, the final analytical sample included 29,225 participants **(**Fig. 1**)**. Of these, 2,829 participants with COPD were identified, corresponding to a weighted prevalence of 9.7%. The weighted baseline characteristic stratified by COPD status are presented in Table 1. Significant differences between participants with and without COPD were observed for age, sex, race/ethnicity, marital status, education level, PIR, smoking status, alcohol consumption, BMI, hypertension, diabetes, cardiovascular disease, asthma, total energy intake, and TAC quartiles (all *P* < 0.05), whereas no significant difference was found for total physical activity (*P* = 0.468).

**Fig. 1:**
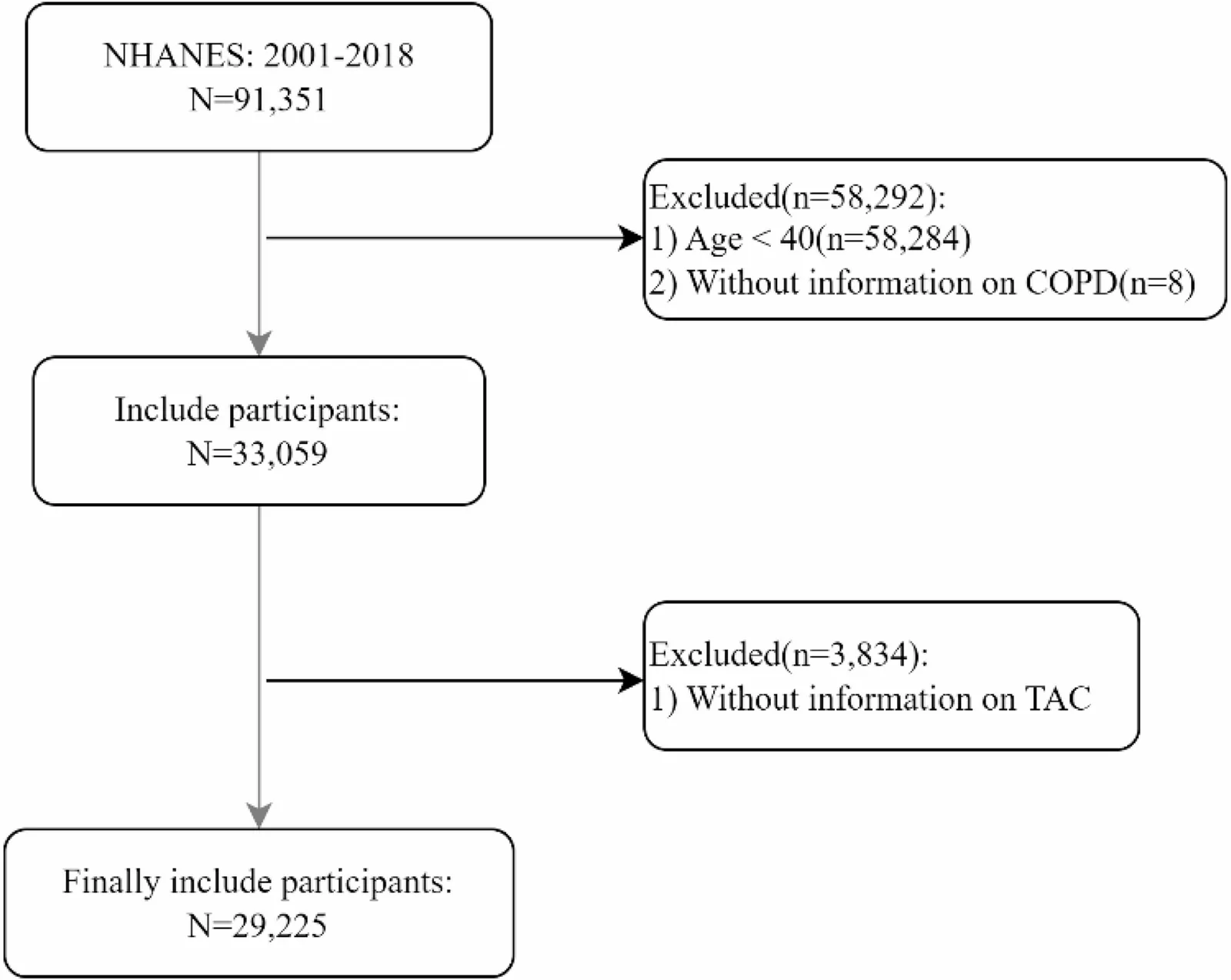
Flow chart of the selection strategy

**Table 1:** Characteristics of the study participants

Characteristic	*N*	Overall (*n* = 29,225)	Non-COPD (*n* = 26,396)	COPD (*n* = 2,829)	*P*-value
Age	29,225	57.53 ± 11.94	57.17 ± 11.91	60.87 ± 11.79	< 0.001
Sex	29,225				< 0.001
Male		14,346 (47.39%)	13,122 (48.31%)	1,224 (38.76%)	
Female		14,879 (52.61%)	13,274 (51.69%)	1,605 (61.24%)	
Ethnicity	29,225				< 0.001
Non-Hispanic White		13,863 (73.64%)	12,099 (72.92%)	1,764 (80.42%)	
Non-Hispanic Black		6,202 (10.19%)	5,684 (10.37%)	518 (8.51%)	
Mexican American		4,404 (5.85%)	4,213 (6.23%)	191 (2.28%)	
Other Hispanic		2,403 (4.34%)	2,215 (4.49%)	188 (3.01%)	
Other Race		2,353 (5.98%)	2,185 (6.00%)	168 (5.78%)	
Marital status	29,206				< 0.001
Married/Living with Partner		18,138 (67.94%)	16,689 (69.03%)	1,449 (57.72%)	
Widowed/Divorced/Separated		8,792 (25.21%)	7,636 (24.11%)	1,156 (35.50%)	
Never married		2,276 (6.85%)	2,054 (6.85%)	222 (6.78%)	
Education level	29,185				< 0.001
Below high school		4,025 (6.54%)	3,667 (6.48%)	358 (7.07%)	
High school		10,963 (34.80%)	9,716 (33.86%)	1,247 (43.65%)	
Above high school		14,197 (58.66%)	12,974 (59.66%)	1,223 (49.28%)	
Poverty income ratio	26,747				< 0.001
Low		7,546 (17.40%)	6,532 (16.26%)	1,014 (28.09%)	
Middle		10,304 (34.98%)	9,238 (34.21%)	1,066 (42.21%)	
High		8,897 (47.62%)	8,366 (49.54%)	531 (29.70%)	
Smoking	29,201				< 0.001
Never		14,728 (50.89%)	13,925 (53.18%)	803 (29.48%)	
Former		9,048 (30.88%)	7,933 (30.22%)	1,115 (36.97%)	
Now		5,425 (18.24%)	4,514 (16.59%)	911 (33.54%)	
Drinking	26,910				< 0.001
Never		4,050 (11.47%)	3,743 (11.58%)	307 (10.41%)	
Former		5,970 (18.31%)	5,190 (17.30%)	780 (27.84%)	
Mild		9,719 (40.47%)	8,924 (41.25%)	795 (33.04%)	
Moderate		3,510 (15.61%)	3,183 (15.74%)	327 (14.45%)	
Heavy		3,661 (14.14%)	3,314 (14.13%)	347 (14.26%)	
BMI	28,650	29.29 ± 6.58	29.16 ± 6.40	30.59 ± 7.94	< 0.001
Hypertension	29,218				< 0.001
No		12,712 (49.55%)	11,793 (50.83%)	919 (37.63%)	
Yes		16,506 (50.45%)	14,596 (49.17%)	1,910 (62.37%)	
Diabetes	29,213				< 0.001
No		24,074 (86.72%)	21,975 (87.65%)	2,099 (78.05%)	
Yes		5,139 (13.28%)	4,412 (12.35%)	727 (21.95%)	
CVD	29,221				< 0.001
No		24,383 (86.70%)	22,516 (88.47%)	1,867 (70.29%)	
Yes		4,838 (13.30%)	3,876 (11.53%)	962 (29.71%)	
PA	19,974	3,148.36 ± 5,053.38	3,154.65 ± 5,065.70	3,080.20 ± 4,918.67	0.468
Asthma	29,197				< 0.001
No		25,499 (86.96%)	23,860 (89.94%)	1,639 (59.11%)	
Yes		3,698 (13.04%)	2,516 (10.06%)	1,182 (40.89%)	
Total energy intake	29,225	2,038.44 ± 829.51	2,049.99 ± 834.08	1,930.76 ± 777.59	< 0.001
TAC group	29,225				< 0.001
Q1		7,307 (24.37%)	6,396 (23.59%)	911 (31.62%)	
Q2		7,306 (25.93%)	6,572 (25.80%)	734 (27.11%)	
Q3		7,306 (25.52%)	6,683 (25.93%)	623 (21.79%)	
Q4		7,306 (24.18%)	6,745 (24.69%)	561 (19.48%)	

Continuous variables are presented as mean ± standard deviation (Mean ± SD), and categorical variables are presented as unweighted counts and percentages [n (%)]. Group comparisons for continuous variables were performed using the design-based Kruskal–Wallis test, and comparisons for categorical variables were performed using Pearson’s chi-square test with Rao–Scott adjustment to account for the complex survey design

*Abbreviations  BMI *body mass index, *CVD *cardiovascular disease, *PIR *poverty-to-income ratio, *TAC *dietary total antioxidant capacity, *PA *physical activity, *Q1–Q4* represent TAC quartiles 1–4 (lowest to highest)

### Association between dietary TAC and COPD

In the unadjusted model (Model 1), compared with the lowest TAC quartile (Q1), higher TAC quartiles were progressively associated with lower odds of COPD: Q2 (OR = 0.78, 95% CI: 0.68–0.91; *P* = 0.001), Q3 (OR = 0.63, 95% CI: 0.54–0.73; *P* < 0.001), and Q4 (OR = 0.59, 95% CI: 0.51–0.68; *P* < 0.001). After adjusting for demographic and socioeconomic factors—including age, sex, race/ethnicity, marital status, education level, and PIR (Model 2)—the inverse association persisted: Q2 (OR = 0.81, 95% CI: 0.69–0.94; *P* = 0.007), Q3 (OR = 0.65, 95% CI: 0.55–0.77; *P* < 0.001), and Q4 (OR = 0.68, 95% CI: 0.58–0.79; *P* < 0.001). In the fully adjusted model (Model 3), additionally controlling for smoking status, alcohol consumption, BMI, comorbidities (hypertension, diabetes, cardiovascular disease, and asthma), total physical activity, total energy intake, and pack-years of smoking, the inverse association between higher TAC and COPD risk remained significant for the upper quartiles: Q2 (OR = 0.83, 95% CI: 0.66–1.02; *P* = 0.084), Q3 (OR = 0.71, 95% CI: 0.57–0.88; *P* = 0.003), and Q4 (OR = 0.78, 95% CI: 0.62–0.99; *P* = 0.040) (Table 2). The McFadden-like pseudo-R² and Tjur R² values for Models 1–3 are presented in Supplementary Table S1. For the fully adjusted Model 3, the corresponding values were 0.5534 and 0.1587, respectively.

**Table 2:** Multivariable logistic regression results for the association of TAC quartiles with COPD

Characteristic	Model 1			Model 2			Model 3		
	OR	95% CI	*P*-value	OR	95% CI	*P*-value	OR	95% CI	*P*-value
TAC group									
Q1	—	—		—	—		—	—	
Q2	0.78	0.68, 0.91	0.001	0.81	0.69, 0.94	0.007	0.83	0.66, 1.02	0.084
Q3	0.63	0.54, 0.73	< 0.001	0.65	0.56, 0.77	< 0.001	0.71	0.57, 0.88	0.003
Q4	0.59	0.51, 0.68	< 0.001	0.68	0.58, 0.79	< 0.001	0.78	0.62, 0.99	0.040
P for trend			< 0.001			< 0.001			0.100

Model 1: unadjusted. Model 2: adjusted for age, sex, ethnicity, marital status, education level, poverty income ratio. Model 3: Model 2 + smoking status, drinking, BMI, hypertension, diabetes, cardiovascular disease, asthma, total physical activity, total energy intake, and cumulative smoking exposure (pack-years). P for trend was derived by modeling the median TAC value of each quartile as a continuous variable

Notably, while individual quartile comparisons (Q3 and Q4 vs. Q1) remained statistically significant in the fully adjusted model, the P for linear trend across quartiles was 0.100, not reaching conventional significance. This attenuation is consistent with the nonlinear dose–response pattern identified by the RCS analysis, in which COPD risk decreased steeply at low-to-moderate TAC levels but plateaued at higher levels. A monotonic linear trend test may therefore underestimate the strength of the association when the true relationship is curvilinear. The significant overall association in the RCS analysis (*P* = 0.005).

### Dose-response relationship

RCS analyses revealed a significant nonlinear inverse dose-response relationship between natural log-transformed TAC (lnTAC) and COPD risk across all models (overall association: Model 1, *P* < 0.001; Model 2, *P* < 0.001; Model 3, *P* = 0.005) **(**Fig. 2, Figure S1–S2). The test for nonlinearity did not reach statistical significance in the crude model (*P* = 0.101), but was statistically significant in Model 2 (*P* = 0.013) and Model 3 (*P* = 0.024). The fitted curve for the fully adjusted model (Model 3, Fig. 2**)** indicated that COPD risk decreased rapidly as lnTAC increased at low-to-moderate levels, but this risk reduction plateaued at higher lnTAC values. Clinically, this plateau suggests diminishing marginal benefits at higher dietary TAC levels, although the wider confidence intervals at the extremes indicate greater uncertainty regarding the precise shape of the association.

**Fig. 2:**
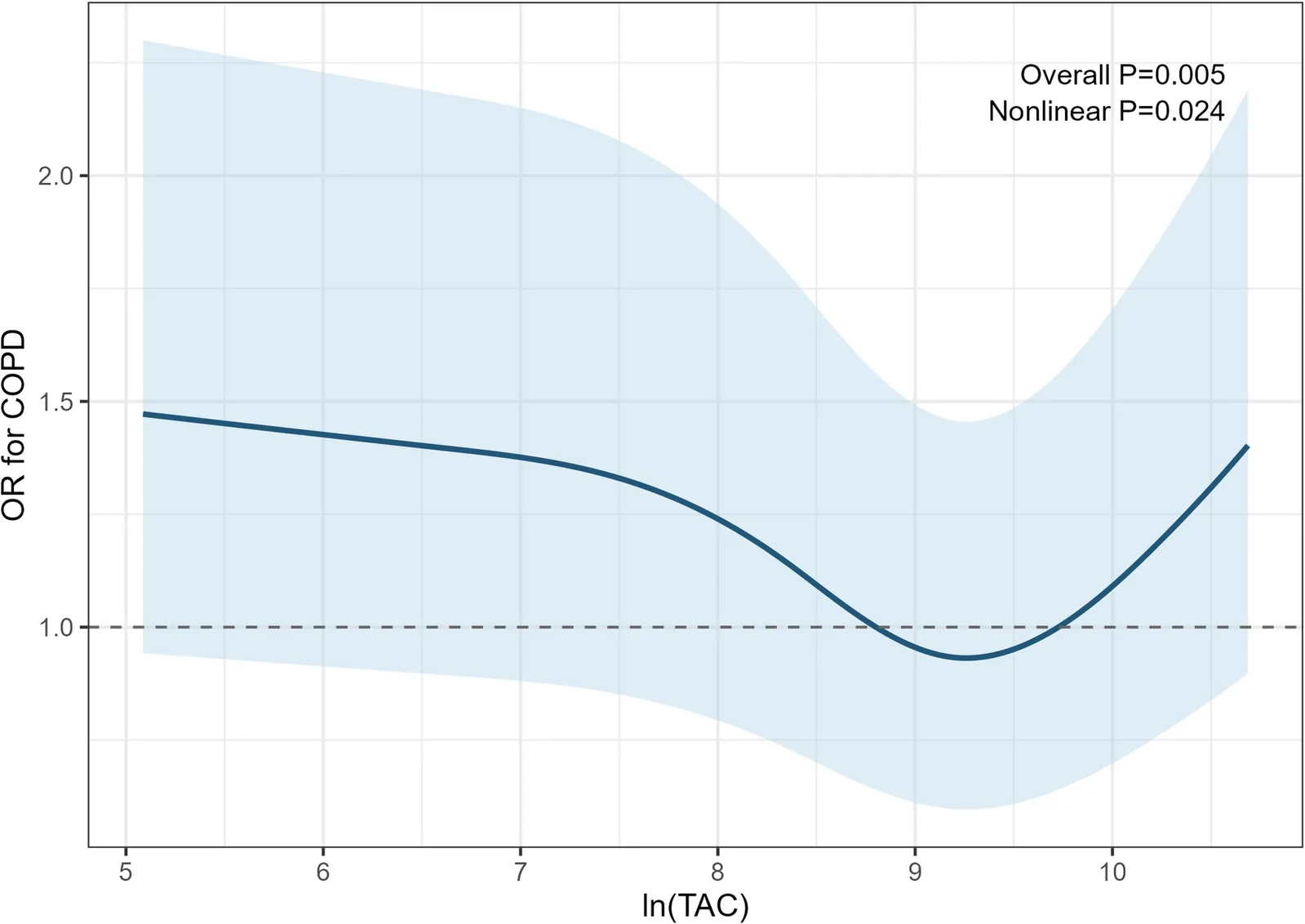
Restricted cubic spline analysis of the dose-response relationship between log-transformed TAC and COPD (Model 3)

### Subgroup analysis and effect modification

Subgroup analyses were performed across multiple variables, including age, sex, race/ethnicity, marital status, education level, PIR, smoking status, drinking status, and common chronic conditions (hypertension, diabetes, cardiovascular disease, and asthma) **(**Fig. 3**)**. In the overall analysis using Model 3, each 1-unit increase in lnTAC was associated with a 8% lower odds of COPD (OR = 0.92, 95% CI: 0.86–0.98; *P* = 0.014). No statistically significant effect modifications were observed across any of the stratification variables: age (P for interaction = 0.975), sex (P for interaction = 0.792), race/ethnicity (P for interaction = 0.095), marital status (P for interaction = 0.558), education level (P for interaction = 0.122), PIR (P for interaction = 0.093), drinking status (P for interaction = 0.544), hypertension (P for interaction = 0.315), diabetes (P for interaction = 0.088), cardiovascular disease (P for interaction = 0.383), and asthma (P for interaction = 0.181). These findings suggest that the inverse association between dietary TAC and COPD is generally consistent across the examined subgroups after adjustment for smoking intensity.

**Fig. 3:**
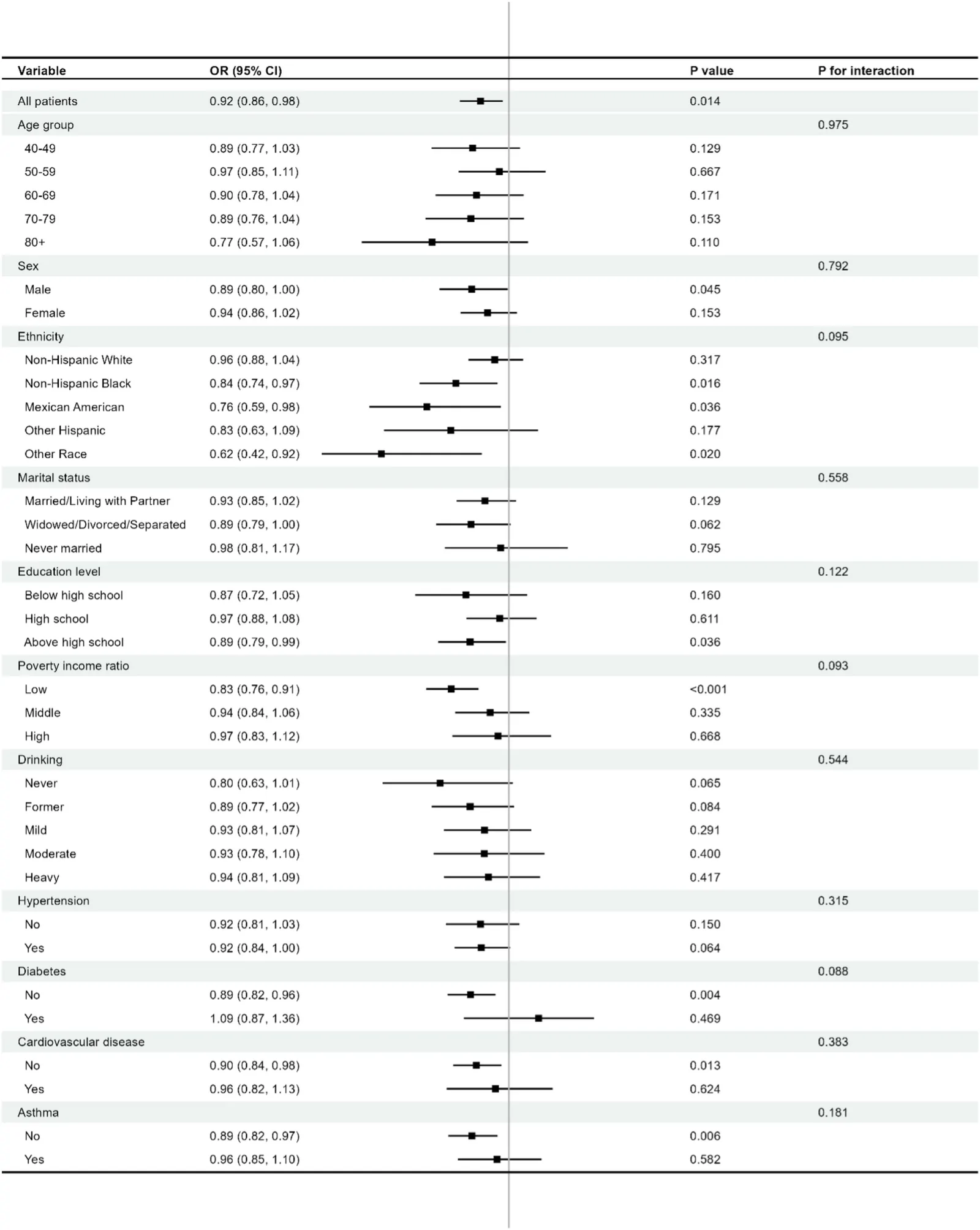
Subgroup analyses of the association between ln TAC (per unit increase) and COPD (Model 3)

### Missingness assessment and sensitivity analyses

A comparison of baseline characteristics between participants with and without available TAC data revealed significant differences in race/ethnicity, education level, poverty income ratio, smoking status, BMI, and cardiovascular disease (all *P* < 0.05), but no significant difference in COPD status (*P* = 0.592) (Supplementary Table S2). To evaluate the robustness of the primary results to potential selection bias from TAC data missingness and covariate missingness, two sensitivity analyses were performed. In the IPW sensitivity analysis, the main findings were preserved: Q3 (OR = 0.68, 95% CI: 0.55–0.85; *P* < 0.001) and Q4 (OR = 0.74, 95% CI: 0.58–0.93; *P* = 0.011) remained significantly associated with lower COPD risk compared with Q1 (Supplementary Table S3). The IPW analysis was conducted on a slightly larger sample (*N* = 16,940) before the additional exclusion of participants with missing pack-years data required for the fully adjusted Model 3. In the multiple imputation analysis, Q3 (OR = 0.82, 95% CI: 0.69–0.97; *P* = 0.021) and Q4 (OR = 0.82, 95% CI: 0.70–0.97; *P* = 0.020) also showed consistent inverse associations (Supplementary Table S4). These findings indicate that the primary results are robust to potential biases arising from missing data.

## Discussion

In this large, nationally representative cross-sectional study of U.S. adults, we found a significant inverse association between higher dietary TAC and the prevalence of COPD. This association remained robust after extensive adjustment for demographic, socioeconomic, lifestyle, clinical, and smoking intensity (pack-years) factors, and exhibited a nonlinear dose-response pattern. After full adjustment, the inverse association was broadly consistent across all examined subgroups, with no statistically significant effect modification detected.

The primary finding is consistent with the oxidative stress hypothesis of COPD pathogenesis [4], which posits that an imbalance between oxidants and antioxidants plays a key role in disease development and progression. Unlike the majority of previous studies that focused on individual antioxidant nutrients—such as vitamin C, vitamin D, or flavonoids—which have reported inconsistent and sometimes contradictory results, our investigation employed TAC as a composite measure of overall dietary antioxidant exposure. This integrative approach captures the synergistic and cumulative effects of multiple dietary antioxidants, thereby offering a more comprehensive and physiologically relevant perspective on antioxidant protection in relation to COPD. Prior research on single antioxidants has yielded mixed findings, contributing to ongoing debate in the field. For example, studies examining vitamin C have reported both protective associations, such as a 12–18% reduction in COPD risk in some cohorts, and null findings in others. Similarly, investigations into vitamin D status and COPD have been inconsistent, with some suggesting a protective role for sufficient serum levels, while others observed no significant effect [21–23]. In contrast, studies using a TAC approach—though fewer in number—have generally shown more consistent relationships with respiratory outcomes. Our findings are in line with this growing body of evidence indicating that higher dietary TAC is associated with improved lung health. For example, Varraso et al. [24] reported that higher cured meat intake was associated with an increased risk of COPD in men, supporting the potential relevance of pro-oxidant dietary exposures to COPD development, although dietary TAC was not directly assessed. Similarly, Hanson et al. [25] found that higher serum tocopherol (vitamin E) levels were associated with better lung function in older adults, further supporting the role of antioxidants in respiratory health. Additionally, an analysis by Walda et al. [26] indicated that a diet high in fruit and vegetables—key contributors to TAC—was associated with reduced COPD mortality in European men. The longitudinal cohort study demonstrated that higher dietary fiber intake, particularly fruit fiber, from childhood through adolescence was consistently associated with improved lung function—specifically higher FEV₁ and FVC—in early adulthood. Increased fiber consumption was also linked to a lower prevalence of asthma with wheeze and a reduced risk of persistent wheeze over time, suggesting a protective role of fiber, possibly mediated through gut-lung axis mechanisms such as short-chain fatty acid production and systemic anti-inflammatory effects [27]. More recently, Bentley et al. [28] specifically examined dietary antioxidants and lung function decline in older adults and reported that higher antioxidant intake was linked to a slower decline in FEV1.

An earlier NHANES analysis also corroborates our results, demonstrating that each unit increase in dietary TAC was associated with a 2.1% increase in FEV1 and a 1.8% increase in FVC [20, 28]. However, not all studies have reached the same conclusion. For example, a European cohort study by Cook et al. [18] found no meaningful relationship between dietary patterns rich in antioxidants and COPD risk after full adjustment (HR = 1.02, 95% CI: 0.91–1.15). Additionally, a case-control study by Martinez et al. [19] reported no significant differences in dietary antioxidant intake between individuals with and without undiagnosed obstructive lung disease.

These discrepancies may stem from several methodological and population-related factors, including differences in TAC assessment methods, variations in the definition and diagnosis of COPD (self-report vs. spirometry), heterogeneity in study populations (e.g., age, smoking status, geographic region), and residual confounding from unmeasured lifestyle or environmental exposures. Despite these inconsistencies, the overall evidence from TAC-focused studies—including our own—suggests that a diet high in diverse antioxidants may confer greater protective benefits against COPD than any single nutrient alone. This highlights the importance of adopting an integrated dietary perspective in both future research and public health recommendations related to respiratory disease prevention [26–29] .

The observed nonlinear dose-response relationship suggests a potential threshold or a saturation effect. The steepest reduction in COPD risk occurred with moderate increases in TAC, with diminishing benefits at very high TAC levels. This pattern may be biologically plausible because the marginal effects of dietary antioxidants may diminish once an adequate antioxidant level is reached, although the precise underlying mechanism remains uncertain [30, 31].

Subgroup analyses using the fully adjusted model (Model 3) showed no statistically significant effect modification across any of the examined strata, including age, sex, race/ethnicity, education, income, drinking status, and common comorbidities (all P for interaction > 0.05). The absence of significant interactions after adjustment for smoking intensity suggests that cumulative smoking exposure, when adequately controlled, does not differentially modify the TAC–COPD association across subgroups. Nonetheless, several strata showed point estimates below 1.0 with borderline significance, warranting further investigation in larger samples.

The potential mechanisms linking higher TAC to lower COPD risk are multifaceted. Antioxidants can directly scavenge reactive oxygen species (ROS) generated from inhaled pollutants and cigarette smoke, reducing oxidative damage to lung lipids, proteins, and DNA [32]. They may also modulate inflammatory pathways (e.g., NF-κB, MAPK), leading to decreased production of pro-inflammatory cytokines (e.g., IL-8, TNF-α) and attenuation of the neutrophilic inflammation characteristic of COPD [33, 34]. Furthermore, antioxidants can protect against protease-mediated elastin degradation and enhance the activity of endogenous antioxidant enzymes (e.g., superoxide dismutase, glutathione peroxidase) [35, 36].

Our findings carry significant clinical and public health implications. They reinforce the importance of a diet rich in antioxidants, which are abundant in fruits, vegetables, whole grains, nuts, and tea, as part of a holistic strategy for COPD prevention and management [37, 38]. This finding aligns with dietary patterns like the Mediterranean diet, which is associated with higher TAC and reduced respiratory disease risk [39, 40]. Healthcare providers should consider incorporating nutritional counseling that focuses on overall dietary quality and antioxidant-rich foods, especially for high-risk individuals such as smokers.

The strengths of this study include the use of a large, nationally representative sample, a composite measure of TAC, comprehensive adjustment for potential confounders, and sophisticated statistical methods accounting for the complex survey design. However, several limitations should be acknowledged. First, the cross-sectional design precludes causal inference and cannot rule out reverse causality, whereby existing COPD may reduce dietary intake and lower TAC. Second, dietary TAC was estimated from a single 24-hour recall, which may not reflect habitual intake and is subject to measurement error and day-to-day variability [41]. Third, COPD status was based on self-reported physician diagnosis rather than objective spirometry, which may lead to misclassification of COPD status. Self-report tends to underestimate mild or undiagnosed COPD, potentially diluting the observed association. While spirometry-based definitions would be preferable, self-reported physician-diagnosed COPD has been validated in prior studies [19]. Fourth, NHANES does not provide data on the duration or severity of COPD (e.g., GOLD stage), nor on the temporal relationship between dietary intake and disease onset. Future studies using longitudinal designs with spirometry-confirmed COPD and detailed disease phenotyping are needed to validate these findings. Fifth, despite extensive adjustment, residual confounding by unmeasured or imperfectly measured factors, such as occupational exposures, air pollution, and genetic predisposition, cannot be excluded. Sixth, the findings are derived from a U.S. population and may not be generalizable to other populations with different dietary patterns, lifestyles, and environmental exposures. Moreover, while TAC provides an integrated measure of antioxidant potential, it does not distinguish the specific contributions or interactions of individual antioxidant compounds, which may have distinct biological roles in COPD pathogenesis. Seventh, although the primary analysis used a complete-case approach, sensitivity analyses using inverse probability weighting and multiple imputation yielded consistent results, mitigating concerns about bias from missing covariate data.

Future research should employ longitudinal or cohort designs to establish temporality and examine the relationship between changes in dietary TAC and COPD incidence or progression. Mechanistic studies are needed to elucidate the specific biological pathways involved. Investigations into the synergistic effects of different dietary antioxidants and the role of TAC in specific COPD phenotypes, such as frequent exacerbators, would be valuable. Furthermore, intervention trials are warranted to test whether dietary modifications aimed at increasing TAC can improve lung function and clinical outcomes in individuals with or at risk for COPD.

## Conclusion

This cross-sectional study of U.S. adults provides evidence that higher dietary TAC is associated with a lower prevalence of COPD. It exhibits a nonlinear dose-response relationship. This protective association is consistent across population subgroups after adjustment for smoking intensity. These findings underscore the potential importance of overall dietary antioxidant intake in the context of COPD risk and support the integration of dietary strategies emphasizing antioxidant-rich foods into public health recommendations for respiratory disease prevention. Prospective studies are needed to confirm these associations, and to explore underlying biological mechanisms.

## Supplementary Information

Supplementary Material 1.

Supplementary Material 2.

Supplementary Material 3.

## Data Availability

All data generated or analysed during this study are included in this published article.

## Abbreviations

(TAC): Total antioxidant capacity
(COPD): Chronic obstructive pulmonary disease
(RCS): Restricted cubic spline
(NCHS): National Center for Health Statistics
