# Supplementary material for: Association between dietary total antioxidant capacity and chronic obstructive pulmonary disease: findings from the national health and nutrition examination survey
Source: BMC Pulm Med. 2026 Aug 13;26:559. doi: 10.1186/s12890-026-04595-3 (PMC13761095; doi:10.1186/s12890-026-04595-3)
Supplement: Supplementary file 1 [file 12890_2026_4595_MOESM3_ESM.docx]

**Figure Legends of Supplementary figures**

**Figure S1** Restricted cubic spline analysis of the dose-response relationship between log-transformed TAC and COPD (crude model).

**Figure S2** Restricted cubic spline analysis of the dose-response relationship between log-transformed TAC and COPD (Model 2).

**Supplementary Table S1: Pseudo-R² values for logistic regression models**

| **Model** | **McFadden-like pseudo-R²** | **Tjur R²** |
| --- | --- | --- |
| Model 1 | 0.0061 | 0.0039 |
| Model 2 | 0.1239 | 0.0370 |
| Model 3 | 0.5534 | 0.1587 |

McFadden-like pseudo-R² summarizes the improvement in the weighted log-likelihood relative to the null model. Tjur R² is the difference in mean predicted COPD probability between cases and non-cases.

**Supplementary Table S2: Comparison of baseline characteristics between participants with and without available TAC data**

| **Variable** | **TAC available (n = 29,225)** | **TAC missing (n = 3,834)** | **P value** |
| --- | --- | --- | --- |
| Age | 57.53 ± 0.14 | 58.60 ± 0.43 | 0.167 |
| Sex: Male | 14,346 (47.39%) | 1,775 (44.87%) | 0.109 |
| Sex: Female | 14,879 (52.61%) | 2,059 (55.13%) |  |
| Ethnicity: Non-Hispanic White | 13,863 (73.64%) | 1,511 (56.18%) | <0.001 |
| Ethnicity: Non-Hispanic Black | 6,202 (10.19%) | 805 (14.72%) |  |
| Ethnicity: Mexican American | 4,404 (5.85%) | 537 (8.08%) |  |
| Ethnicity: Other Hispanic | 2,403 (4.34%) | 344 (5.56%) |  |
| Ethnicity: Other Race | 2,353 (5.98%) | 637 (15.45%) |  |
| Education: Below high school | 4,025 (6.54%) | 772 (13.31%) | <0.001 |
| Education: High school | 10,963 (34.80%) | 1,478 (36.48%) |  |
| Education: Above high school | 14,197 (58.66%) | 1,543 (50.22%) |  |
| PIR: Low | 7,546 (17.40%) | 1,075 (28.77%) | <0.001 |
| PIR: Middle | 10,304 (34.98%) | 1,158 (32.61%) |  |
| PIR: High | 8,897 (47.62%) | 871 (38.62%) |  |
| Smoking: Never | 14,728 (50.89%) | 2,068 (52.78%) | <0.001 |
| Smoking: Former | 9,048 (30.88%) | 1,056 (24.29%) |  |
| Smoking: Current | 5,425 (18.24%) | 695 (22.93%) |  |
| BMI | 29.29 ± 0.07 | 28.48 ± 0.21 | <0.001 |
| COPD: No | 26,396 (90.31%) | 3,474 (90.73%) | 0.592 |
| COPD: Yes | 2,829 (9.69%) | 360 (9.27%) |  |
| Hypertension: No | 12,712 (49.55%) | 1,841 (49.46%) | 0.950 |
| Hypertension: Yes | 16,506 (50.45%) | 1,979 (50.54%) |  |
| Diabetes: No | 24,074 (86.72%) | 3,147 (85.02%) | 0.090 |
| Diabetes: Yes | 5,139 (13.28%) | 678 (14.98%) |  |
| CVD: No | 24,383 (86.70%) | 3,048 (84.23%) | 0.012 |
| CVD: Yes | 4,838 (13.30%) | 786 (15.77%) |  |
| Asthma: No | 25,411 (86.56%) | 3,370 (87.38%) | 0.452 |
| Asthma: Yes | 3,814 (13.44%) | 464 (12.62%) |  |
| PA total MET | 3,148.36 ± 64.87 | 2,962.72 ± 197.89 | 0.839 |

Data are presented as weighted mean ± SE or n (weighted %). P values from design-based Kruskal–Wallis test (continuous) or Rao–Scott chi-square test (categorical). CVD, cardiovascular disease; PA, physical activity; MET, metabolic equivalent.

**Supplementary Table S3: Inverse probability weighting sensitivity analysis (Model 3)**

| **Characteristic** | **OR** | **95% CI** | **P value** |
| --- | --- | --- | --- |
| Q1 | — | — |  |
| Q2 | 0.79 | 0.64, 0.97 | 0.028 |
| Q3 | 0.68 | 0.55, 0.85 | <0.001 |
| Q4 | 0.74 | 0.58, 0.93 | 0.011 |

Stabilized inverse probability weights were calculated using baseline covariates to predict TAC data availability and multiplied by the NHANES sampling weights. The model was adjusted for the same covariates as Model 3.

**Supplementary Table S4: Multiple imputation sensitivity analysis (Model 3)**

| **Characteristic** | **OR** | **95% CI** | **P value** |
| --- | --- | --- | --- |
| Q1 | — | — |  |
| Q2 | 0.95 | 0.80, 1.11 | 0.507 |
| Q3 | 0.82 | 0.69, 0.97 | 0.021 |
| Q4 | 0.82 | 0.70, 0.97 | 0.020 |

Multiple imputation (m = 20) was used to handle missing covariate data; the COPD outcome and TAC exposure were not imputed. The model was adjusted for the same covariates as Model 3.
